# Thermal Accumulation and Collagen Remodeling in Porcine Models: Parameter‐Dependent Efficacy of Micro‐Focused Ultrasound for Human Facial Skin Tightening

**DOI:** 10.1111/jocd.70719

**Published:** 2026-02-11

**Authors:** Jing Qi, Bo Wei, Jing Pan, Haiping Zhang

**Affiliations:** ^1^ Department of Dermatology Xuan Wu Hospital, Capital Medical University Beijing China; ^2^ Hunan JIANFLASER Laser Medical Technology Co., Ltd. Changsha Hunan Province China; ^3^ Hunan Provincial Drug Evaluation and Adverse Reaction Monitoring Center Changsha Hunan Province China

**Keywords:** clinical efficacy, dose‐effect relationship, facial tightening, micro‐focused ultrasound, porcine skin tissue

## Abstract

**Background:**

Skin laxity, driven by collagen/elastin degradation and aging, is targeted by micro‐focused ultrasound (MFU) through depth‐specific thermal coagulation to induce collagen remodeling, yet parameter‐dependent thermal kinetics and safety thresholds remain underexplored, limiting protocol optimization.

**Aims:**

To validate the dose‐effect relationship of MFU using in vitro porcine skin tissues and evaluate its safety and efficacy for human facial skin tightening.

**Methods:**

Porcine skin tissues containing intact skin, fat, and muscle layers were treated with 8D‐DL 3.0, 8D‐DL 4.5, Vmax‐DL 3.0, and Vmax‐DL 4.5 handpieces. Subcutaneous temperatures at depths of 2.0 mm, 3.0 mm, 4.5 mm, and 6.0 mm were recorded under varying parameters (energy levels and exposure durations). Twenty patients undergoing single‐session MFU facial treatment between June and August 2024 were enrolled. Skin tightening outcomes were assessed at 30‐ and 90‐days post‐treatment.

**Results:**

For 8D‐DL 3.0 and Vmax‐DL 3.0 handpieces, peak temperatures occurred at 3.0 mm depth across all energy levels and exposure durations. For 8D‐DL 4.5 and Vmax‐DL 4.5 handpieces, peak temperatures localized at 4.5 mm. Focal depth temperatures increased significantly with higher energy levels and prolonged exposure. At 30‐ and 90‐days post‐treatment, upward displacement and volume reduction in bilateral cheek and jawline regions were observed. Mild procedural pain was reported, with no adverse events.

**Conclusion:**

MFU‐induced thermal peaks align with preset focal depths, demonstrating parameter‐dependent thermal accumulation. The procedure safely achieves clinically significant facial skin tightening.

## 1 Introduction

Skin laxity, a hallmark of aging, results from collagen and elastin degradation exacerbated by intrinsic senescence and extrinsic factors like photoaging. Non‐invasive skin‐tightening technologies, including micro‐focused ultrasound (MFU), have gained traction due to their minimal downtime and favorable safety profiles [1]. MFU employs high‐frequency ultrasound (4–10 MHz) with low energy density (0.4–1.2 J/cm^2^) to thermally target the reticular dermis (1.5–3.0 mm) and superficial musculoaponeurotic system (SMAS, 4.5 mm), inducing immediate collagen denaturation and subsequent neocollagenesis through controlled thermal injury (60°C–70°C) [2]. While studies validate its depth‐specific coagulative effects [3], the dose–response relationship—specifically, how energy parameters (e.g., subcutaneous depth, energy levels, exposure duration) influence thermal kinetics and spatial precision—remains poorly characterized, limiting protocol optimization [4].

Safety and efficacy in MFU hinge on balancing thermal accumulation with tissue‐specific tolerance. Prior reports confirm focal temperatures > 60°C at predefined depths (e.g., 3.0 or 4.5 mm), correlating with collagen remodeling and skin retraction [5]. However, excessive energy delivery risks adverse events, such as transient nerve irritation or subcutaneous fat atrophy, particularly in high‐risk zones like the periorbital region [6]. Unlike high‐intensity focused ultrasound (HIFU), MFU's shorter pulse durations (50–200 ms) and lower energy flux minimize collateral damage, making it suitable for delicate facial anatomy [7]. Despite its advantages, systematic evaluations of thermal distribution across multilayered tissue models—critical for validating device calibration and minimizing off‐target effects—are lacking, underscoring the need for translational ex vivo studies [8].

This study employs ex vivo porcine skin (structurally analogous to human skin in collagen density, dermal thickness, and thermal conductivity) to quantify thermal gradients at subcutaneous depths (2.0–6.0 mm) using 8D‐DL and Vmax‐DL handpieces. Concurrently, 20 patients undergoing MFU facial treatment are assessed for skin tightening (via 3D volumetric analysis) and adverse events at 30/90‐day follow‐ups. By correlating laboratory‐derived thermal profiles (peak temperatures at 3.0/4.5 mm, aligning with device presets) with clinical outcomes, this dual‐method approach establishes a mechanistic dose–response framework, ensuring both safety and efficacy in aesthetic practice.

## 2 Materials and Methods

### 2.1 Study Design and Ethical Approval

This dual‐phase study combined ex vivo porcine tissue experiments with a prospective clinical trial to evaluate the dose–response relationship and clinical efficacy of MFU for skin tightening. The protocol was approved by the Ethics Committee of Xuan Wu Hospital, Capital Medical University (Approval No. MDER‐2024‐004‐002), and conducted in accordance with the Declaration of Helsinki.

### 2.2 Ex Vivo Porcine Skin Study

#### 2.2.1 Specimen Preparation

Fresh porcine skin specimens (10 × 10 cm) containing intact epidermis, dermis, subcutaneous fat, and superficial muscle layers were procured from a certified abattoir. Specimens were stored at 4°C in phosphate‐buffered saline (PBS)‐moistened gauze and used within 24 h to preserve tissue integrity.

#### 2.2.2 Experimental Setup

Thermocouple Placement: T‐type thermocouples (accuracy ±0.5°C; Omega Engineering) were inserted perpendicularly at subcutaneous depths of 2.0 mm, 3.0 mm, 4.5 mm, and 6.0 mm using a sterile depth‐calibrated guide needle.

Treatment Parameters: Four handpieces were tested:

8D‐DL 3.0 and Vmax‐DL 3.0 (focal depth: 3.0 mm, frequency: 4 MHz).

8D‐DL 4.5 and Vmax‐DL 4.5 (focal depth: 4.5 mm, frequency: 4 MHz).

Each handpiece was applied at three energy levels (I: 0.4 W, III: 2.0 W, V: 3.5 W) and three exposure durations (30 s, 150 s, 300 s), with three replicates per parameter combination (total 108 treatments). Energy titration began at Level I (0.4 W) with stepwise 0.5‐W increments after each test pulse. Escalation continued until patients reported tolerable discomfort (VAS 4–6/10), not exceeding Level V (3.5 W). Final energy levels were maintained uniformly across treatment zones. Ultrasound gel (Parker Laboratories) ensured acoustic coupling.

#### 2.2.3 Temperature Monitoring

Real‐time thermal data were recorded at 1‐s intervals using a 16‐channel thermocouple logger (TES‐1310, TES Electrical Electronic Corp.). Peak temperatures at each depth were analyzed to determine thermal accumulation trends.

### 2.3 Clinical Trial on Human Facial Skin

#### 2.3.1 Participant Selection

Twenty patients (age ≥ 18 years; Fitzpatrick skin types II–IV) with mild‐to‐moderate facial laxity were enrolled between June and August 2024. Inclusion criteria included avoidance of concurrent aesthetic treatments (e.g., fillers, lasers, surgery) for 6 months. Exclusion criteria encompassed active facial infections, pregnancy, or systemic comorbidities.

#### 2.3.2 Treatment Protocol

Pre‐Treatment Preparation: Patients were positioned supine, with treatment zones (mid/lower face, jawline) demarcated using a surgical marker.

Energy Delivery: The Model JF‐CS Series MFU system (Jianfeng Medical Technology) equipped with 8D‐DL 3.0, 8D‐DL 4.5, Vmax‐DL 3.0, and Vmax‐DL 4.5 handpieces was applied. Treatment parameters followed a standardized protocol:

Pulse frequency: 5–10 Hz (single‐point mode).

Energy titration: Adjusted per patient tolerance (I–V energy levels).

Overlap: 10%–15% between pulses to ensure uniform coverage.

#### 2.3.3 Outcome Measures

Three‐dimensional volumetric analysis was performed using Vectra H2 3D imaging system (Canfield Scientific) at baseline, 30 and 90 days. The mid‐lower facial and jawline regions were segmented using bony and soft‐tissue landmarks (Figure S1), with displacement vectors calculated from canthus‐commissure intersections. Displacement (mm) quantified upward movement of the jawline contour relative to a fixed reference plane (Frankfort horizontal). Negative displacement values indicate downward tissue sagging relative to baseline (e.g., −0.5 mm = 0.5 mm inferior displacement). All reported values represent net upward tightening (positive) or relapse (negative). Volume reduction (cc) was calculated using Mirror software via mesh deformation analysis of pre‐defined regions of interest (ROIs: bilateral midface, jawline, submental).

Patient‐Reported Outcomes: Pain Assessment: Visual Analog Scale (VAS; 0 = no pain, 10 = worst pain) immediately post‐treatment.

Efficacy Evaluation: Global Aesthetic Improvement Scale (GAIS) scored by blinded assessors at 30/90 days (1 = worsened, 5 = very much improved).

Satisfaction Survey: 5‐point Likert questionnaire (1 = very dissatisfied, 5 = very satisfied) at 90 days.

Safety Monitoring: Adverse events (erythema, edema, dyspigmentation) were documented throughout the study.

### 2.4 Statistical Analysis

Data analysis was performed using SPSS 26.0 (IBM Corp.). Continuous variables with normal distribution were expressed as mean ± SD; non‐parametric data as median (IQR). Comparisons between baseline, 30‐day, and 90‐day outcomes used paired *t*‐tests (parametric) or Wilcoxon signed‐rank tests (non‐parametric). Temperature trends across energy levels and durations were analyzed using general linear models with Bonferroni correction. Categorical variables (GAIS, satisfaction) were assessed via chi‐square tests. Two‐sided *p* < 0.05 was considered as statistical significance.

## 3 Results

### 3.1 Ex Vivo Porcine Skin Study

#### 3.1.1 Focal Temperature Distribution

**FIGURE 1:**
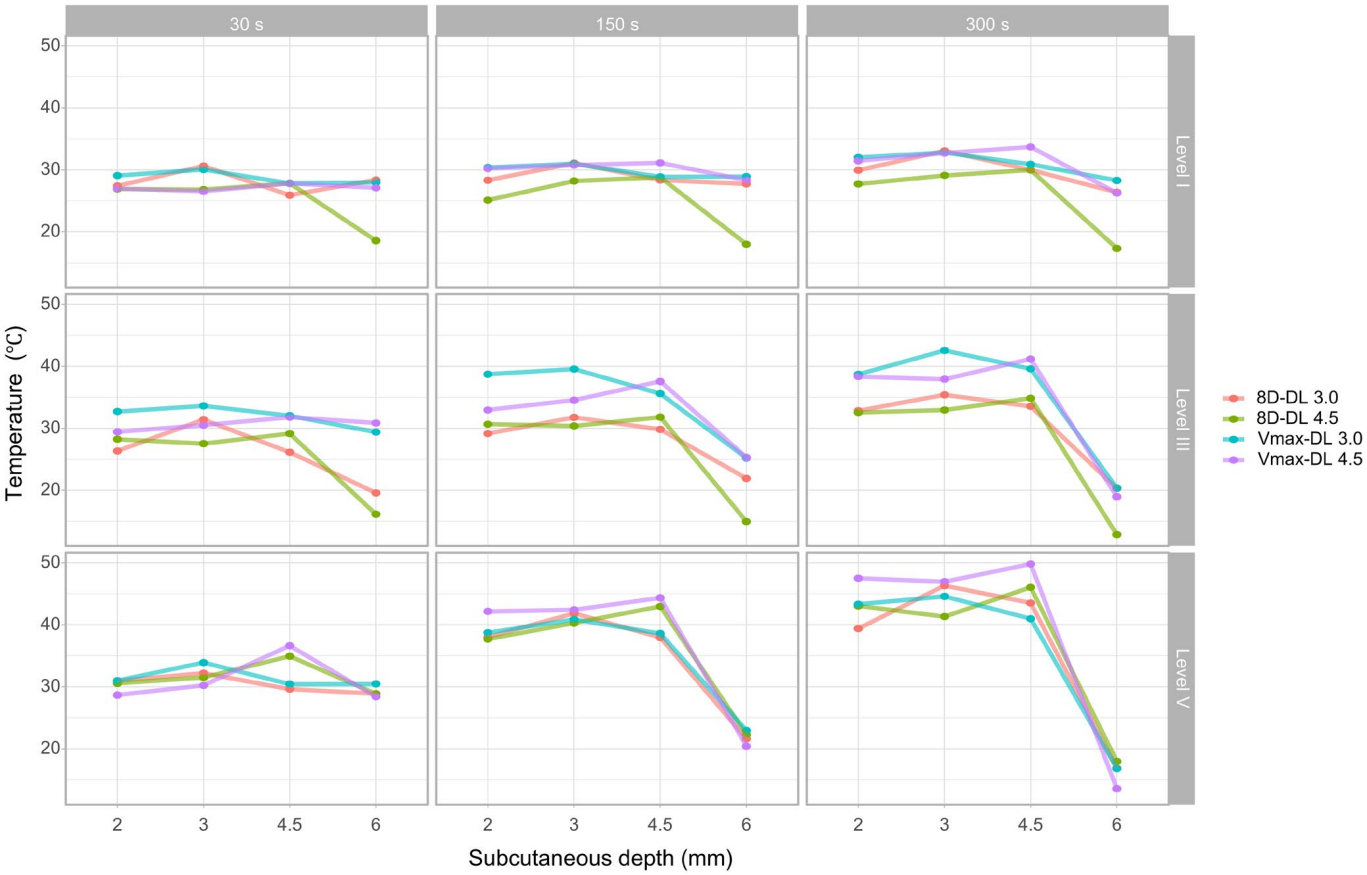
Temperature variations in ex vivo porcine skin tissue across treatment handpieces, energy levels, and exposure durations.

The peak temperatures generated by the 8D‐DL 3.0 and Vmax‐DL 3.0 handpieces consistently localized at the 3.0 mm subcutaneous depth across all energy levels (I, III, V) and exposure durations (30, 150, 300 s). Similarly, the 8D‐DL 4.5 and Vmax‐DL 4.5 handpieces achieved maximal thermal accumulation at the 4.5 mm depth, aligning with their preset focal targets (Figure 1).

#### 3.1.2 Dose‐Dependent Thermal Accumulation

**FIGURE 2:**
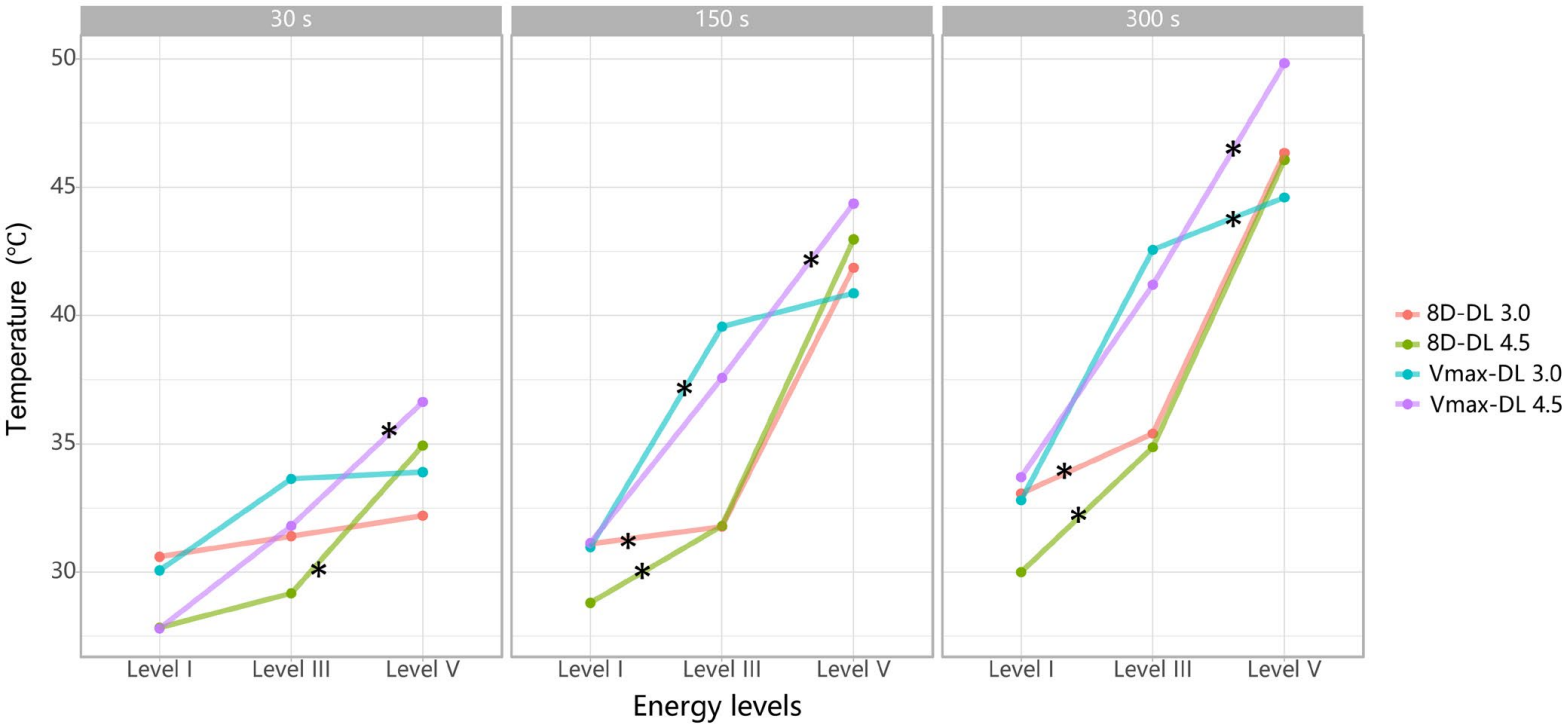
Temperature dependence on energy levels. ^*^ *p* < 0.05.

**FIGURE 3:**
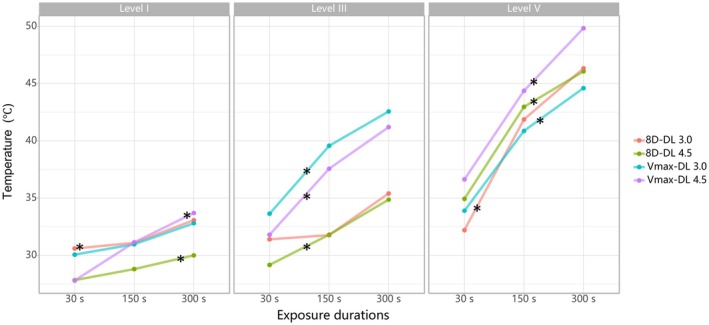
Temperature dependence on exposure durations. ^*^ *p* < 0.05.

Temperature at the focal depth (3.0 or 4.5 mm) exhibited an overall positive correlation with both energy levels and exposure durations, with only minor variations observed across experimental groups (Figures 2, 3). For example, at 300 s exposure duration, the 8D‐DL 3.0 handpiece increased focal temperature from 33.1°C (level I) to 46.3°C (level V) at 3.0 mm depth, while the 8D‐DL 4.5 handpiece elevated temperatures from 30.0°C (level I) to 46.1°C (level V) at 4.5 mm (Figure 2). Additionally, at energy level V, the 8D‐DL 3.0 handpiece increased focal temperature from 32.2°C (30 s) to 46.3°C (300 s) at 3.0 mm depth, while the 8D‐DL 4.5 handpiece elevated temperatures from 34.9°C (30 s) to 46.1°C (300 s) at 4.5 mm (Figure 3).

### 3.2 Clinical Trial on Human Facial Skin

#### 3.2.1 Demographics and Baseline Characteristics

The study enrolled 20 patients (19 females, 1 male; mean age 38.05 ± 5.72 years) with Fitzpatrick skin types II (*n* = 1) and III (*n* = 19). Body mass index (BMI) remained stable throughout the study (baseline: 24.11 ± 4.08; 30 days: 24.12 ± 4.03; 90 days: 24.50 ± 3.95 kg/m^2^; *p* = 0.9423) (Table 1).

**TABLE 1:** Baseline characteristics of patients.

	0 day	30 days post‐treatment	90 days post‐treatment	F‐value	*p*
Age [$\bar{x}\pm s$, years]	38.05 ± 5.72				
Gender [n (%)]					
Male	1 (5.0%)				
Female	19 (95.0%)				
Skin type [n (%)]					
Type II	1 (5.0%)				
Type III	19 (95.0%)				
Height [$\bar{x}\pm s$, cm]	164.85 ± 3.72	164.85 ± 3.72	164.85 ± 3.72	0	1.00
Weight [$\bar{x}\pm s$, kg]	65.72 ± 12.79	65.80 ± 12.64	66.82 ± 12.37	0.05	0.9535
BMI [$\bar{x}\pm s$, kg/m^2^]	24.11 ± 4.08	24.12 ± 4.03	24.50 ± 3.95	0.06	0.9423

Abbreviation: BMI, Body Mass Index.

#### 3.2.2 Efficacy Outcomes

Skin Tightening: At 30 days post‐treatment, significant reductions in skin laxity were observed across all regions: Mid/lower face: Left side displacement = 0.56 (0.10, 1.12) mm, volume reduction = −1.57 (−2.70, −0.31) cc; Right side displacement = 0.50 (0.30, 1.18) mm, volume reduction = −1.71 (−3.02, −0.15) (all *p* < 0.01 vs. baseline). Jawline: Left side displacement = 1.58 (0.16, 3.14) mm, volume reduction = −1.29 (−5.34, 0.81); Right side displacement = 1.18 (0.49, 2.71) mm, volume reduction = −2.06 (−8.01, −0.58) (all *p* < 0.05 vs. baseline).

**FIGURE 4:**
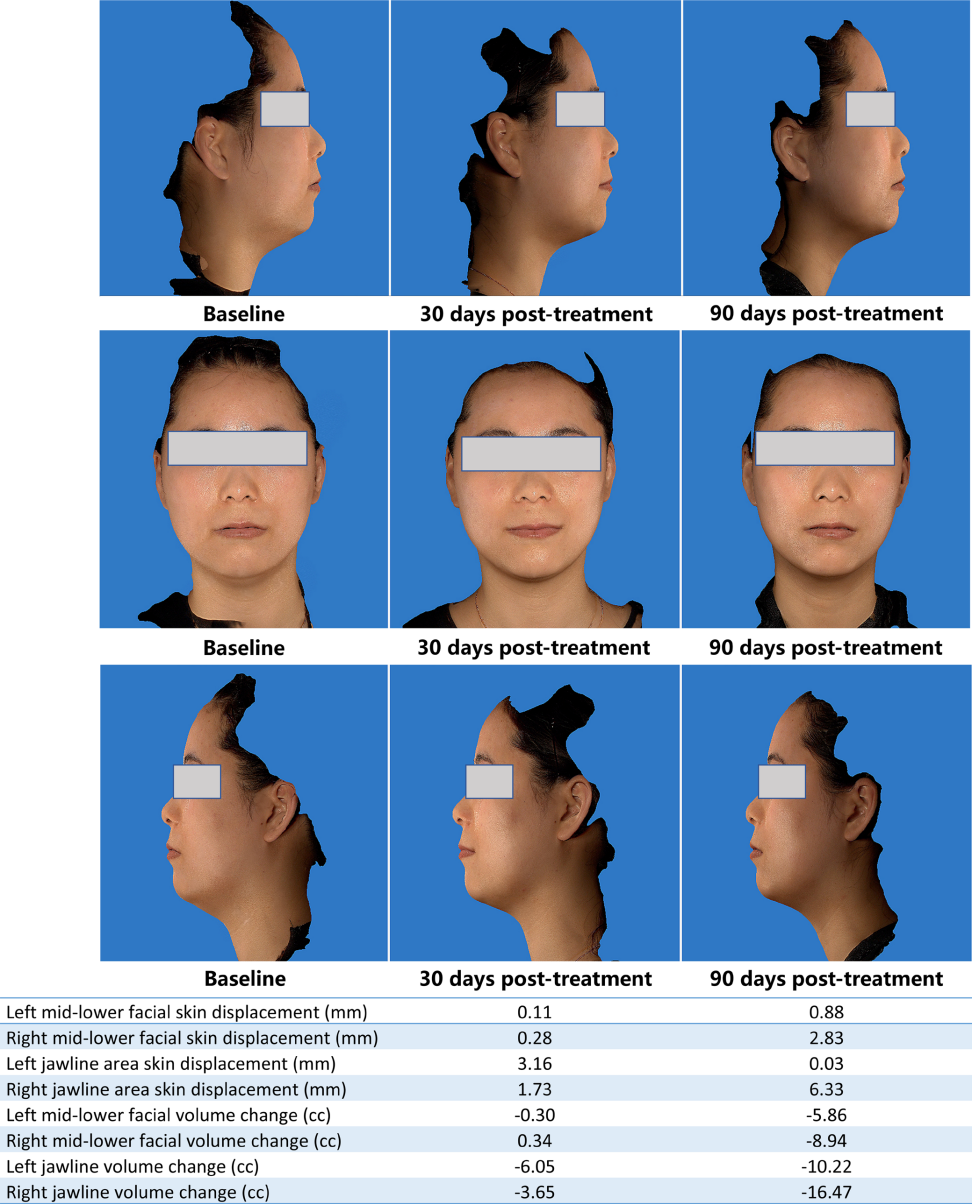
Representative case 1: Female, 31 years.

**FIGURE 5:**
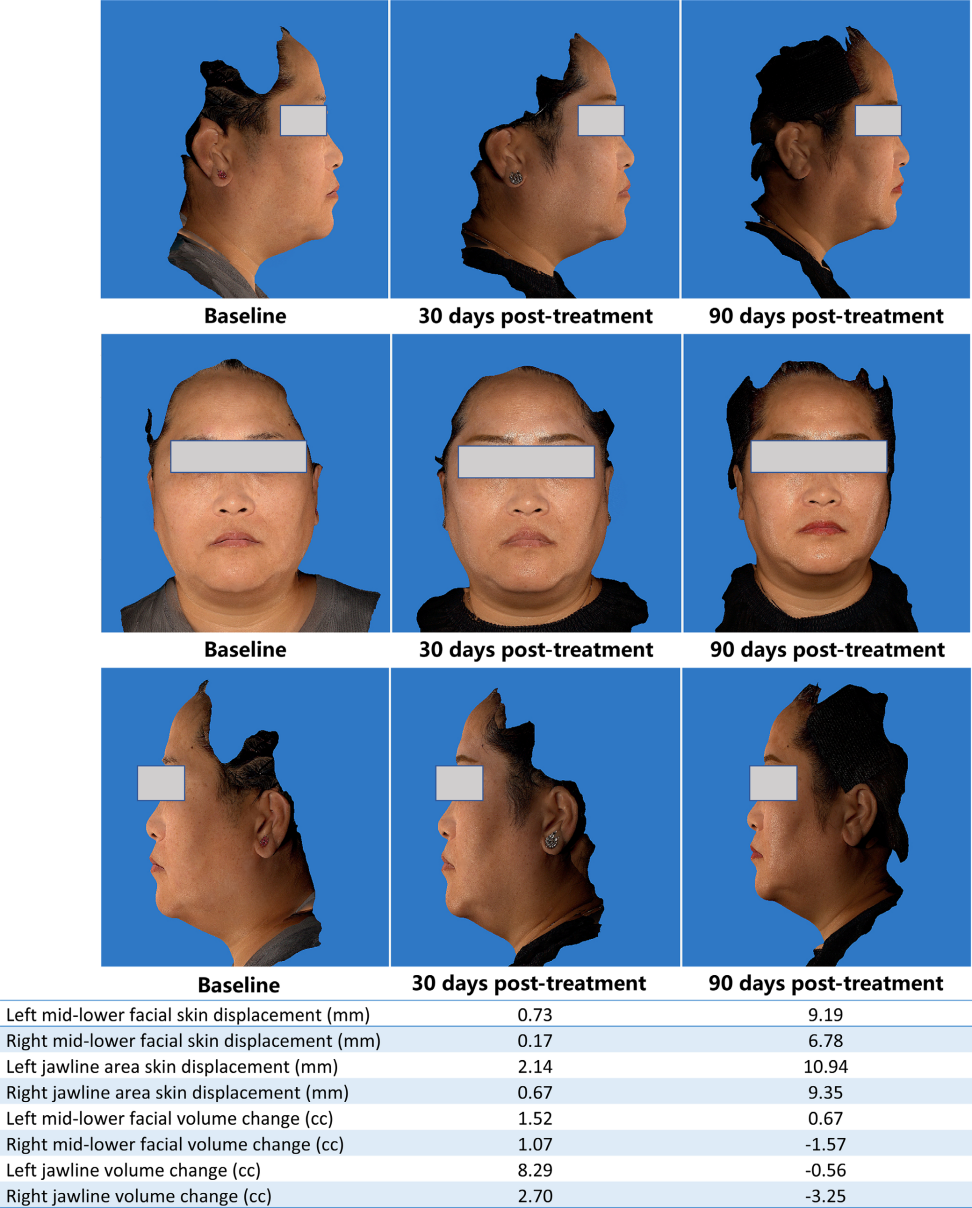
Representative case 2: Female, 35 years.

Improvements plateaued at 90 days, with no significant differences between 30‐ and 90‐day outcomes (all *p* > 0.05). Median GAIS scores improved from baseline to 30 days (2.00 [IQR: 1.00–2.00]) and further by 90 days (1.50 [IQR: 1.00–2.00]), reflecting sustained aesthetic enhancement at 90 days; 75% (15/20) of patients reported “very satisfied” and 25% (5/20) “satisfied” on the Likert scale (Table 2, and Figures 4, 5).

**TABLE 2:** Comparison of efficacy parameters at 30‐ and 90‐days post‐treatment (median [Q1, Q3]).

	30 days post‐treatment	*S*‐value	*p*	90 days post‐treatment	*S*‐value	*p*	30 vs. 90 days post‐treatment	
							*S*‐value	*p*
Left mid‐lower facial skin displacement (mm)	0.56 (0.10, 1.12)	80	0.0017	0.35 (0.08, 0.96)	66	0.0121	−18	0.5217
Right mid‐lower facial skin displacement (mm)	0.50 (0.30, 1.18)	91	0.0002	0.43 (0.05, 1.35)	72	0.0056	−22	0.4304
Left jawline area skin displacement (mm)	1.58 (0.16, 3.14)	89	0.0003	1.44 (0.56, 3.73)	94	0.0001	22	0.4304
Right jawline area skin displacement (mm)	1.18 (0.49, 2.71)	100	< 0.0001	1.55 (0.38, 5.19)	78	0.0023	23	0.4091
Left mid‐lower facial volume change (cc)	−1.57 (−2.70, −0.31)	−86	0.0006	−2.51 (−3.57, −0.99)	−91	0.0002	−16.50	0.5520
Right mid‐lower facial volume change (cc)	−1.71 (−3.02, −0.15)	−82	0.0012	−3.00 (−5.45, −0.91)	−84	0.0009	−37.00	0.1769
Left jawline volume change (cc)	−1.29 (−5.34, 0.81)	−53	0.0484	−2.85 (−7.23, −0.17)	−67	0.0107	−39.00	0.1536
Right jawline volume change (cc)	−2.06 (−8.01, −0.58)	−66	0.0121	−2.27 (−12.45, −0.39)	−69	0.0083	−35.00	0.2024
Investigator GAIS score	2.00 (1.00, 2.00)			1.50 (1.00, 2.00)			−5.00	0.3125
Subject GAIS score	2.00 (1.00, 2.00)			1.50 (1.00, 2.00)			−5.50	0.2500
Patient satisfaction								
4 points				5 (25%)				
5 points				15 (75%)				

*Note:* Non‐parametric data (Wilcoxon signed‐rank test) reported as Median (Q1, Q3). Negative *S*‐values indicate downward trends.

Abbreviation: GAIS, Global Aesthetic Improvement Scale.

#### 3.2.3 Safety Profile

Pain Assessment: Mean VAS score during treatment was 3.64 ± 1.99, indicating mild, tolerable discomfort.

Adverse Events: One patient (5%) reported transient, mild pain localized to the jawline, resolving spontaneously within 20 days without intervention. No cases of erythema, edema, or dyspigmentation were observed.

## 4 Discussion

MFU represents a paradigm shift in non‐invasive skin tightening by leveraging the unique properties of ultrasound waves—deep tissue penetration and spatial precision—to induce controlled thermal injury in the dermis and superficial musculoaponeurotic system (SMAS) [1]. As demonstrated in this study, MFU at 4 MHz with energy densities of 0.4–3.5 J/cm^2^ achieves focal temperatures sufficient for collagen denaturation (> 60°C) at preset depths (3.0 and 4.5 mm), aligning with its proposed mechanism of neocollagenesis and immediate tissue contraction [9, 10]. While earlier applications of MFU in oncology and pain management emphasized ablative precision [11], its adaptation to aesthetic medicine has been constrained by a lack of robust dose–response data and objective efficacy metrics [12, 13]. Our findings bridge this gap by systematically correlating thermal kinetics with clinical outcomes, offering insights into both mechanistic and practical dimensions.

The ex vivo porcine model revealed critical thermal dynamics: peak temperatures at 3.0 mm (8D‐DL 3.0 and Vmax‐DL 3.0) and 4.5 mm (8D‐DL 4.5 and Vmax‐DL 4.5) corresponded precisely to handpiece presets, validating the device's spatial accuracy [9]. Although absolute temperatures in ex vivo tissue (max 46.3°C) fell below the 60°C threshold required for complete collagen denaturation in vivo, this discrepancy is mechanistically explained by the absence of vascular perfusion and metabolic activity in ex vivo models, which critically mitigate heat dissipation through convective blood flow [14]. Evidence confirms perfusion increases thermal diffusivity by ~53% in live tissues compared to ex vivo systems, creating intrinsic limitations for replicating in vivo thermoablative thresholds [15].

Importantly, collagen denaturation represents a progressive transition beginning with helix destabilization at ~40°C and culminating in complete structural transition at 61.5°C [16]. Thus, sub‐denaturation temperatures in our model still demonstrate a robust linear relationship between energy parameters (energy, duration) and thermal accumulation, underscoring MFU's dose‐dependent behavior [4]. The translational rationale for ex vivo‐to‐vivo extrapolation further derives from conserved structural properties: porcine and human skin exhibit closely matched collagen density, epidermal thickness, and extracellular matrix composition [17]. These parallels validate the model's predictive utility for focal targeting despite thermal amplitude differences. Consequently, while ex vivo temperatures are inherently conservative due to unmodifiable hemodynamic factors, the model's spatial accuracy and thermal distribution patterns remain clinically informative for in vivo applications [16].

Clinically, MFU elicited statistically significant improvements in mid/lower face and jawline laxity at 30 days. These outcomes persisted at 90 days, suggesting sustained collagen remodeling rather than transient edema—a common confounder in energy‐based therapies [4]. The integration of 3D volumetric analysis (Vectra H2) provided objective, quantifiable metrics, addressing a key limitation of prior studies reliant on subjective scales like GAIS [5]. Patient satisfaction rates further validate MFU's efficacy, particularly in addressing submental and jawline contouring—a therapeutic niche where traditional modalities often underdeliver [18].

Notably, the reduced pulse width (22–25 ms) of the Model JF‐CS Series MFU system minimized thermal diffusion, mitigating risks of subcutaneous fat atrophy and neurovascular injury associated with longer pulses (50–100 ms) in conventional HIFU [19]. This innovation likely contributed to the favorable safety profile: only one case (5%) of transient jawline pain resolved spontaneously, contrasting with earlier reports of dyspigmentation and prolonged edema in HIFU cohorts [20]. The mean VAS score of 3.64 ± 1.99 reflects enhanced patient tolerability, enabling maximal energy delivery (90% at energy level V) without compromising safety—a critical advantage in aesthetic practice where comfort influences adherence [21].

While our study advances MFU's translational framework, limitations warrant consideration. First, the ex vivo model's inability to replicate dynamic biological responses (e.g., inflammation, angiogenesis) necessitates caution in extrapolating thermal thresholds to clinical settings [17]. Second, although significant tightening persisted at 90 days, collagen maturation typically peaks at 6–12 months post‐treatment [13]. Thus, the 90‐day follow‐up period may not fully capture the peak of collagen maturation, and longer‐term studies are needed to assess durability and late‐onset adverse events. Third, while our clinical cohort (*n* = 20) demonstrated statistically significant efficacy, subgroup analyses (e.g., by skin type or age) were underpowered. Future studies with larger samples are warranted to explore demographic or anatomic variability.

## 5 Conclusion

In conclusion, this dual‐method investigation establishes MFU as a precise, dose‐tunable modality for facial skin tightening, with thermal accumulation governed by energy parameters and focal depth. The congruence between ex vivo thermal profiles and clinical efficacy—coupled with minimal adverse events—supports its adoption as a first‐line non‐surgical option for mild‐to‐moderate skin laxity. Future work should explore combinatorial regimens (e.g., MFU with radiofrequency) to synergize collagen remodeling across dermal and subdermal compartments.

## Author Contributions

Conception of the manuscript was done by Jing Qi and Haiping Zhang. The manuscript was drafted by Jing Qi, substantively edited, and revised by Bo Wei, Jing Qi, and Haiping Zhang.

## Ethics Statement

The protocol was approved by the Ethics Committee of Xuanwu Hospital, Capital Medical University (Approval No. MDER‐2024‐004‐002), and conducted in accordance with the Declaration of Helsinki.

## Conflicts of Interest

The authors declare no conflicts of interest.

## Supporting information


**Figure S1:** Schematic diagram of facial 3D volumetric analysis and measurement areas. This figure illustrates the measurement regions and displacement points for the mid‐lower face and jawline. The mid‐lower facial region measurement area is defined superiorly by the line connecting the alar base, most prominent point of the zygomatic arch, and auricular groove; inferiorly by the mandibular border; and medially by a vertical line 1 cm lateral to the oral commissure. The jawline region measurement area is bounded superiorly by the mandibular border; inferiorly by a horizontal line 1 cm superior to the laryngeal prominence; laterally by an anterior vertical line through the earlobe; and medially by the facial midline. Displacement points include the mid‐facial point located at the intersection of the horizontal line through bilateral oral commissures and vertical line through the lateral canthus, and the jawline point at the intersection of the vertical line through the lateral canthus and horizontal line 1.5–2 cm superior to the laryngeal prominence, both serving as measurement points.

## Data Availability

The data that support the findings of this study are available from the corresponding author upon reasonable request.
