# Supplementary material for: Thermal Accumulation and Collagen Remodeling in Porcine Models: Parameter‐Dependent Efficacy of Micro‐Focused Ultrasound for Human Facial Skin Tightening
Source: J Cosmet Dermatol. 2026 Feb 11;25(2):e70719. doi: 10.1111/jocd.70719 (PMC12894807; doi:10.1111/jocd.70719)
Supplement: Supplementary file 1 — Figure S1: Schematic diagram of facial 3D volumetric analysis and measurement areas. This figure illustrates the measurement regions and displacement points for the mid‐lower face and jawline. The mid‐lower facial region measurement area is defined superiorly by the line connecting the alar base, most prominent point of the zygomatic arch, and auricular groove; inferiorly by the mandibular border; and medially by a vertical line 1 cm lateral to the oral commissure. The jawline region measurement area is bounded superiorly by the mandibular border; inferiorly by a horizontal line 1 cm superior to the laryngeal prominence; laterally by an anterior vertical line through the earlobe; and medially by the facial midline. Displacement points include the mid‐facial point located at the intersection of the horizontal line through bilateral oral commissures and vertical line through the lateral canthus, and the jawline point at the intersection of the vertical line through the lateral canthus and horizontal line 1.5–2 cm superior to the laryngeal prominence, both serving as measurement points. [file JOCD-25-e70719-s001.docx]

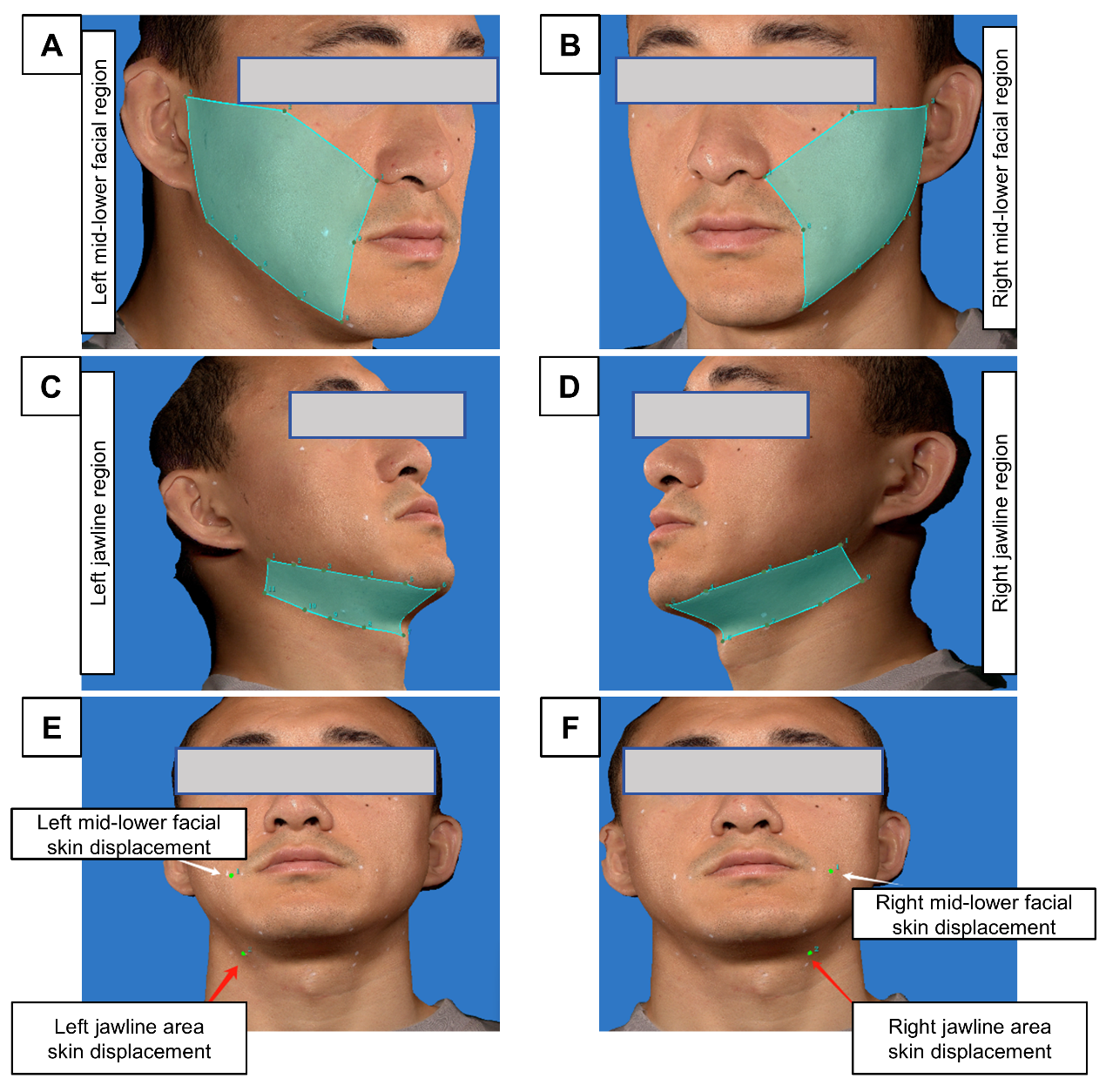


**Figure S1. Schematic diagram of facial 3D volumetric analysis and measurement areas.** This figure illustrates the measurement regions and displacement points for the mid-lower face and jawline. The mid-lower facial region measurement area is defined superiorly by the line connecting the alar base, most prominent point of the zygomatic arch, and auricular groove; inferiorly by the mandibular border; and medially by a vertical line 1 cm lateral to the oral commissure. The jawline region measurement area is bounded superiorly by the mandibular border; inferiorly by a horizontal line 1 cm superior to the laryngeal prominence; laterally by an anterior vertical line through the earlobe; and medially by the facial midline. Displacement points include the mid-facial point located at the intersection of the horizontal line through bilateral oral commissures and vertical line through the lateral canthus, and the jawline point at the intersection of the vertical line through the lateral canthus and horizontal line 1.5-2 cm superior to the laryngeal prominence, both serving as measurement points.
